# Spotlight on gene therapy in Germany

**DOI:** 10.1038/s41434-021-00277-3

**Published:** 2021-09-21

**Authors:** Claudio Mussolino, Patrick Harrison

**Affiliations:** 1 Institute for Transfusion Medicine and Gene Therapy, Medical Center – University of Freiburg, Freiburg, Germany; 2 Faculty of Medicine, University of Freiburg, Freiburg, Germany; 3 Department of Physiology, University College Cork, Cork, Ireland

**Keywords:** Gene therapy, Genetic engineering

Gene therapy has witnessed an explosive growth over the last three decades. Since the first trial in 1990, to treat a rare immunodeficiency due to the lack of adenosine deaminase (ADA), clinical exploitation of diverse gene therapeutics all around the globe has clearly shown the immense potential of curing human diseases with genes. However, the path has not always been plain sailing, and tragic events during this 30- year-long journey, even though limited in number, have led to the development of more cautious approaches and thorough regulatory processes prior to clinical approval. With the inception of precision gene-editing technologies and their first clinical application in 2010, to render T cells resistant to HIV infection, an exciting new era had begun in the field of gene therapy. This culminated in 2020 with the Nobel Prize in Chemistry award going to the two researchers that developed a breakthrough method for precise genome editing, the CRISPR-Cas9. However, 2020 also revealed the potential danger behind the misuse of genome editing technologies with the birth of the first gene-edited humans and subsequently, the prison sentence for the scientist that drove such unlawful application.

Europe is at the forefront of the gene therapy field and major investments in the public and private sectors have propelled the development of novel and more effective therapies to tackle rare diseases and cancer. Efficient modification of patient-derived cells ex vivo either to correct a genetic defect, to replace a missing gene function or to modulate the expression of a protective gene have been attempted with variable success to establish innovative therapeutics for multiple disorders. Cell therapies as potent anti-tumor agents capable of eradicating hematopoietic malignancies have been approved for commercialization. Direct in vivo gene therapy has also recently been authorized to treat certain types of retinal blindness and spinal muscular atrophy. With over 50 clinical studies explored thus far (*source: ClinicalTrials.gov*), Germany is actively involved in developing and testing innovative medicines for the benefit of patients and for the advancement of the gene therapy field. To document this tremendous effort, *Gene Therapy* has commissioned a special issue with submissions from scientists working at German institutions, placing a spotlight on their research efforts which continue to advance this fascinating field from bench to bedside. In this issue, we have reviewed the excellent contribution of research groups located across Germany on a range of topics. These include vector development and clinical exploitation of retroviral gene therapy [1], which continues to be an essential step towards the generation of more effective cell therapeutics. In particular, efficient viral vectors have been a key player in the generation of potent cancer immunotherapeutics based either on T or NK cells [2]. When combined with induced pluripotent stem cell (iPSC) technology or hematopoietic stem cells (HSC), viral vectors can be employed to generate in vitro factories for large-scale production of effector cells amenable to transplantation [3, 4]. This has major implications for the future establishment of novel off-the-shelf cell therapeutics. Despite the unquestioned success achieved in the clinics, a non-trivial genotoxic risk associated with the use of integrating vectors has encouraged the exploration of alternative engineering strategies. This has presented an opportunity for precision genome editing technologies which have expanded dramatically in the last decade in an attempt to develop novel procedures that reduce these risks. On the one hand, transposon-based systems characterized by a semi-random integration profile is approaching the clinic, providing the first evidence that the genome of T cells can be modified to express a chimeric antigen receptor (CAR) using viral-free systems [5]. This could potentially simplify manufacturing and reduce toxicity. On the other hand, precise gene inactivation using designer nucleases based on transcription activator-like effectors or CRISPR-Cas have been used to modify T cells for clinical purposes. In this context, the large-scale production of edited T cells has enabled the clinical exploitation of these modified cells to combat HIV infection [6, 7] or other T cell-related disorders. Similarly, precise genome editing has been applied to provide additional features to CAR T cells with the goal of improving their safety and manufacturing procedures [8]. Since the inception of gene therapy with integrating viral vectors in 1990, we have witnessed an expanding effort to apply these strategies in HSCs to provide a lifelong solution to diseases of the hematopoietic system. To establish innovative therapeutics for a plethora of human genetic defects, HSC modifications using either integrating vectors or precision genome editing technologies have been pursued with variable success. Such procedures are highly challenging because of the heterogeneity that characterizes the target cell population [9]. This certainly highlights that increasing our knowledge of the target cell population is crucial to improve the generation of more effective cell therapeutics in the future. Importantly, gene therapy is not limited to the hematopoietic system. Similar strategies have been devised to tackle devastating human disorders such as muscular dystrophy. In this case, gene replacement and gene-editing strategies are currently being explored in large animal models to provide sufficient evidence that gene therapy may offer new treatment opportunities for these patients [10]. However, the tremendous steps forward also highlight concerns due to the potential immunogenicity of the genome editing components [11] and their potential unwanted activity at off-target sites. This opens new opportunities for the development of strategies to induce immune tolerance towards these editing tools and to develop more precise technologies or novel strategies that avoid DNA cleavage such as base editing, prime editing or ultimately epigenome editing. We believe this spotlight provides a glimpse towards the milestones that have shaped the gene therapy field in the last 30 years and we are grateful to *Gene Therapy* for propelling this initiative and to all the contributing authors for their passion to drive this exciting field.
